# Immune-inflammatory biomarkers and immune features in metabolic dysfunction-associated steatotic liver disease: a bibliometric study

**DOI:** 10.3389/fimmu.2026.1896838

**Published:** 2026-08-12

**Authors:** Zelin Ye, Mingrui Liu, Qingjuan Wu, Jiuchong Wang, Wenliang Lyu

**Affiliations:** 1 Department of Infection, Guang’anmen Hospital, China Academy of Chinese Medical Sciences, Beijing, China; 2 Data Center of Traditional Chinese Medicine, China Academy of Chinese Medical Sciences, Beijing, China

**Keywords:** bibliometric analysis, CiteSpace, immune features, immune-inflammatory biomarkers, liver fibrosis, MASLD, metabolic dysfunction-associated steatotic liver disease, VOSviewer

## Abstract

**Introduction:**

Metabolic dysfunction-associated steatotic liver disease (MASLD) is a heterogeneous liver disease involving metabolic dysfunction, inflammatory activity, immune activation, and fibrotic remodeling. Immune-inflammatory biomarkers and immune-related features may support disease characterization, fibrosis assessment, and risk stratification, but the knowledge structure of this subfield remains unclear. This study used bibliometric methods to map the development, contributors, intellectual structure, and emerging trends of immune-inflammatory biomarker and immune-feature research in MASLD/NAFLD.

**Methods:**

Publications were retrieved from the Web of Science Core Collection (WoSCC), with Scopus used for cross-database validation. After manual screening and data refinement, 268 WoSCC publications from 2000 to 2025 were retained for primary analysis. CiteSpace and VOSviewer were used to analyze publication trends, contributing countries, institutions, journals, authors, collaboration networks, keyword co-occurrence, keyword bursts, and reference co-citation clusters.

**Results:**

Annual publication output increased markedly after 2022. China contributed the largest number of publications, whereas the United States showed the strongest collaborative link strength. Aarhus University was the leading institution, while Henning Grønbæk and Konstantin Kazankov were the leading authors by publication output. Co-citation and keyword analyses indicated that the field is organized around overlapping domains, including systemic inflammatory indices, hepatic inflammation and histologic injury, immune-cell and macrophage-centered features, fibrosis assessment, treatment-response readouts, and prognostic stratification. Recent keyword bursts highlighted oxidative stress, macrophages, pathogenesis, and liver fibrosis. Scopus validation showed broadly consistent temporal and thematic patterns.

**Discussion:**

Overall, this subfield is rapidly expanding toward fibrosis-oriented stratification, immune-cell phenotyping, and clinically scalable inflammatory biomarker research.

## 1 Introduction

Metabolic dysfunction-associated steatotic liver disease (MASLD), formerly known as nonalcoholic fatty liver disease (NAFLD) (1), affecting approximately 30% of the global population (2). Although MASLD is closely related to obesity, insulin resistance, type 2 diabetes, and other cardiometabolic abnormalities (3, 4), its development and progression do not depend solely on metabolic dysfunction. Current evidence shows that hepatocellular lipotoxicity (5), gut-liver crosstalk and microbe-related signals (6), innate and adaptive immune dysregulation (7), immune cell-mediated inflammation (8), and fibrotic remodeling of the hepatic microenvironment (9) in disease progression from simple steatosis to steatohepatitis and fibrosis.

Increasing attention has been paid to immune-inflammatory biomarkers and immune features in MASLD. These outputs include circulating cytokines and chemokines (4), composite systemic inflammatory indices (10, 11), plasma inflammatory protein biomarkers (12), hepatic immune or transcriptomic features (13), circulating immune-cell phenotypes (7), and tissue-level immune features (14). Compared with general metabolic indicators, these immune-related readouts may provide complementary information on inflammatory activity, immune heterogeneity, tissue injury, and fibrotic remodeling. Therefore, they may help to refine disease characterization, fibrosis assessment, mechanistic interpretation, and clinical stratification.

The knowledge structure of this subfield remains unclear. Existing bibliometric studies have mainly focused on NAFLD/MASLD as a whole or adjacent subfields (15, 16), such as the gut-liver axis (17) and macrophage-related research (18), while the bibliometric analysis of the literature specifically targeting immune-inflammatory biomarkers and immune features in MASLD is still limited. Therefore, this study aims to use the Web of Science Core Collection (WoSCC) to provide a bibliometric overview of key literature in this subfield. Specifically, this study analyzed publication trends, major contributing countries, institutions, journals, and authors, and further mapped the knowledge base and temporal evolution of the field through reference co-citation analysis, keyword co-occurrence analysis, and keyword burst detection. This approach was used to clarify the knowledge structure, development trajectory, and emerging research trends of immune-inflammatory biomarkers and immune features in MASLD.

## 2 Methods

### 2.1 Data sources and search strategy

The literature retrieval was conducted in the Web of Science Core Collection (WoSCC) and Scopus databases on May 12, 2026. WoSCC was used as the primary bibliometric database, and Scopus was used as an additional database for multi-database validation. The publication-year range was restricted to 2000–2025. In both databases, document types were restricted to Article and Review, and the language was limited to English. The search strategy was designed to capture studies on MASLD and its historical nomenclature, including metabolic dysfunction-associated steatohepatitis (MASH), nonalcoholic fatty liver disease (NAFLD), and nonalcoholic steatohepatitis (NASH), in combination with terms related to immune-inflammatory biomarkers and immune features. To maintain the focus on non-cancer MASLD/NAFLD and to avoid retrieval drift toward hepatocellular carcinoma, malignancy, and COVID-19-related literature, these terms were excluded at the search stage.

For WoSCC, Topic Search (TS) was used. The final WoSCC search query was as follows: TS=((“metabolic dysfunction-associated steatotic liver disease” OR MASLD OR “metabolic dysfunction-associated steatohepatitis” OR MASH OR “nonalcoholic fatty liver disease” OR NAFLD OR “non-alcoholic fatty liver disease” OR “nonalcoholic steatohepatitis” OR NASH OR “non-alcoholic steatohepatitis”) AND (((immune OR immunolog* OR inflamm* OR cytokine* OR chemokine* OR “immune cell*” OR “immune signature*” OR “immune profil*” OR macrophage* OR “Kupffer cell*” OR monocyte* OR neutrophil* OR lymphocyte* OR “T cell*” OR “B cell*” OR “NK cell*” OR “NKT cell*” OR “dendritic cell*” OR immunoglobulin*) NEAR/3 (biomarker* OR marker* OR signature* OR profil* OR diagnostic* OR prognostic* OR predictor* OR classifier* OR panel* OR score* OR “risk stratification” OR “noninvasive diagnosis” OR “non-invasive diagnosis”)))) NOT TS=(“hepatocellular carcinoma” OR HCC OR cancer* OR tumor* OR tumour* OR carcinoma* OR malignan* OR covid* OR “SARS-CoV-2”). The WoSCC records were downloaded in plain text format with full records and cited references for subsequent screening and bibliometric analysis. For Scopus, search query was as follows: TITLE-ABS-KEY((“metabolic dysfunction-associated steatotic liver disease” OR MASLD OR “metabolic dysfunction-associated steatohepatitis” OR MASH OR “nonalcoholic fatty liver disease” OR NAFLD OR “non-alcoholic fatty liver disease” OR “nonalcoholic steatohepatitis” OR NASH OR “non-alcoholic steatohepatitis”) AND ((immune OR immunolog* OR inflamm* OR cytokine* OR chemokine* OR “immune cell*” OR “immune signature*” OR “immune profil*” OR macrophage* OR “Kupffer cell*” OR monocyte* OR neutrophil* OR lymphocyte* OR “T cell*” OR “B cell*” OR “NK cell*” OR “NKT cell*” OR “dendritic cell*” OR immunoglobulin*) W/3 (biomarker* OR marker* OR signature* OR profil* OR diagnostic* OR prognostic* OR predictor* OR classifier* OR panel* OR score* OR “risk stratification” OR “noninvasive diagnosis” OR “non-invasive diagnosis”))) AND NOT TITLE-ABS-KEY(“hepatocellular carcinoma” OR HCC OR cancer* OR tumor* OR tumour* OR carcinoma* OR malignan* OR covid* OR “SARS-CoV-2”) AND PUBYEAR > 1999 AND PUBYEAR < 2026 AND (DOCTYPE(ar) OR DOCTYPE(re)) AND LANGUAGE(english). The search and record export from both databases were completed on May 12, 2026.

### 2.2 Eligibility criteria and data refinement

Studies were included when MASLD/MASH or NAFLD/NASH was the primary disease context and when the manuscript generated biomarker- or feature-type immune-inflammatory outputs relevant to the present subdomain. Eligible outputs included immune-inflammatory biomarkers, immune profiling, immune signatures, immune infiltration patterns, tissue-level immune characteristics, predictive models, and treatment-responsive immune-inflammatory readouts.

Studies were excluded when MASLD/NAFLD was only a background condition, comorbidity, stratification factor, or secondary context. Studies were also excluded when the main focus was a non-MASLD disease or non-hepatic outcome, or when intervention, exposure, lifestyle, or mechanistic studies lacked biomarker- or feature-type immune-inflammatory outputs.

These general eligibility criteria were operationalized using a prespecified screening codebook comprising eight exclusion rules (E1–E8) and seven eligible study/output categories (I1–I7). Both screeners applied the same codebook during title-and-abstract screening. In the final screening log, each record was assigned one primary eligibility code. Mingrui Liu conducted the initial screening of all 1,595 WoSCC records, and Zelin Ye subsequently verified all records using the same eligibility criteria and codebook. The verified classifications were used to construct the final bibliometric corpus. As a descriptive quality-control measure, percentage agreement and Cohen’s kappa were calculated between the initial and verified binary include/exclude classifications. Record are provided in **Supplementary**.

For the Scopus dataset, records were first cross-matched with the WoSCC retrieval set using DOI and title information. Records overlapping with the WoSCC dataset were used to assess cross-database coverage, whereas Scopus-only records were independently screened using the same eligibility criteria as the WoSCC records.

### 2.3 Bibliometric analysis and visualization

Bibliometric analysis and visualization were performed using CiteSpace (version 7.0.R6) and VOSviewer (version 1.6). VOSviewer (19) was used to construct country, institution, author, and keyword co-occurrence or collaboration networks. In these maps, node size represented the frequency or productivity of an item, link thickness reflected the strength of the relationship between two items, and total link strength (TLS) was used to quantify the overall connectedness of each item within the network. Country, institution, and author analyses were used to describe the geographical and collaborative structure of the field, whereas keyword co-occurrence analysis was used to identify major research hotspots. Journal and author summary tables were generated descriptively: journals were ranked by total citations, and authors were ranked by publication output. Average citations were calculated as total citations divided by the number of documents. Keyword co-occurrence analysis was conducted in VOSviewer using Keywords Plus extracted from the WoSCC records.

CiteSpace (20) was used for reference co-citation clustering, timeline visualization, and keyword burst detection. The time span was set from 2000 to 2025, with a time slice of 1 year. Node selection was based on the g-index with k = 25. Additional CiteSpace parameters were set as follows: link retaining factor (LRF) = 2.5, maximum links per node (L/N) = 10, look-back years (LBY) = 5, and topology pruning coefficient (e) = 1.0. No pruning was applied. Cluster labels were generated using the latent semantic indexing (LSI) method and were used to describe the algorithm-derived co-citation structure. The timeline view was used to display the temporal distribution of co-cited references across clusters. Keyword burst detection was used to identify terms with abrupt increases in citation or keyword attention over time, thereby indicating emerging research fronts.

## 3 Results

### 3.1 Publication output and temporal trends

**Figure 1:**
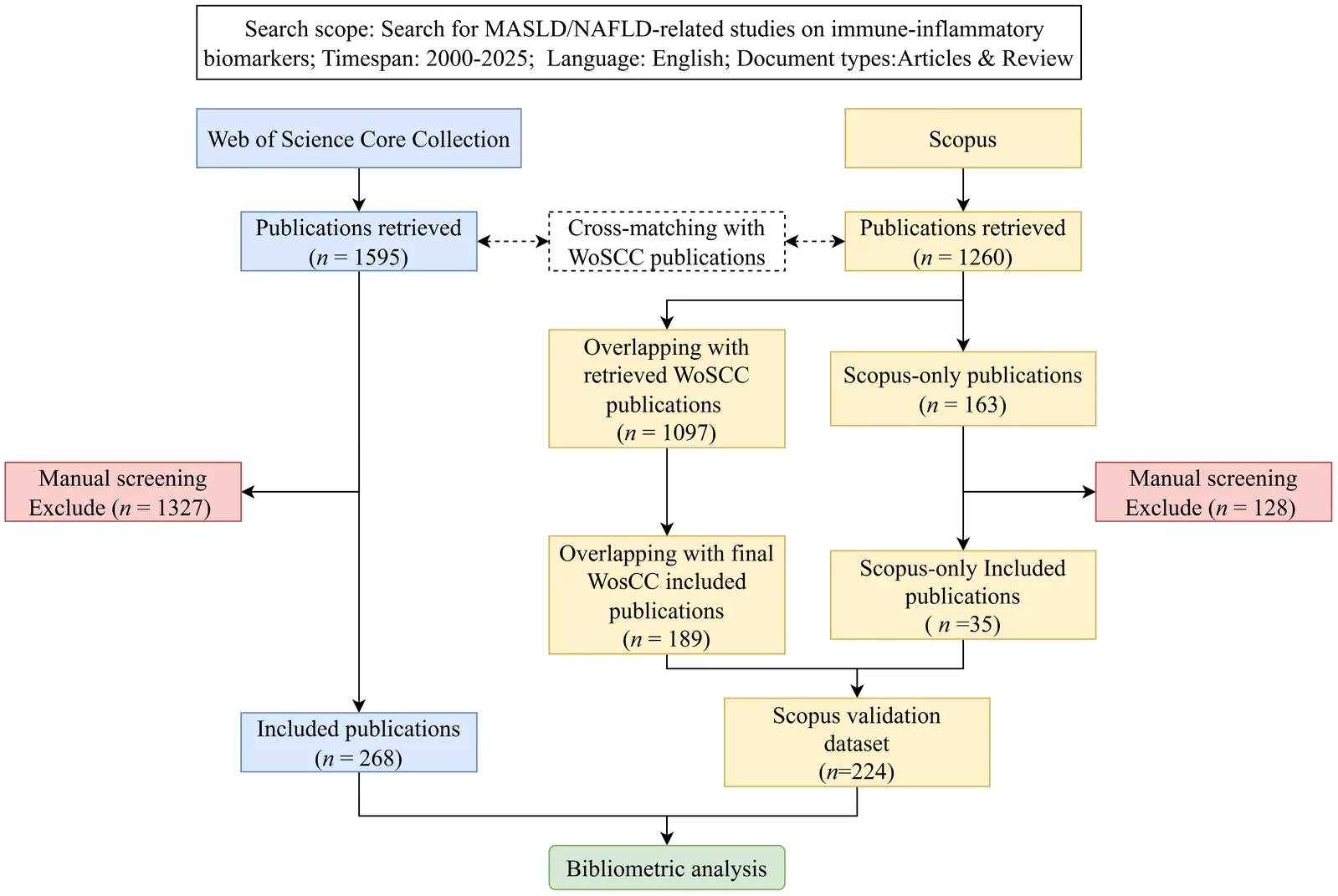
Flowchart of literature retrieval, screening, and cross-database validation. Publications were retrieved from the web of science core collection (WoSCC) and Scopus using search strategies targeting MASLD/NAFLD-related studies on immune-inflammatory biomarkers and immune features. The search was limited to English-language Articles and Reviews published from 2000 to 2025. WoSCC was used as the primary bibliometric dataset: 1,595 records were retrieved, and 268 publications were retained after manual screening and data refinement. Screening was performed at the title-and-abstract level, and the record are provided in **Supplementary Material**. Scopus was used for cross-database validation: 1,260 records were retrieved and cross-matched with WoSCC records using DOI and title information. Among the Scopus records, 189 overlapped with the final WoSCC included publications, and 163 Scopus-only records were further screened using the same eligibility criteria. Thirty-five Scopus-only publications were retained, resulting in a Scopus validation dataset of 224 publications, including 189 overlapping records and 35 Scopus-only eligible records. The full search strategy is provided in Section 2.1.

**Figure 2:**
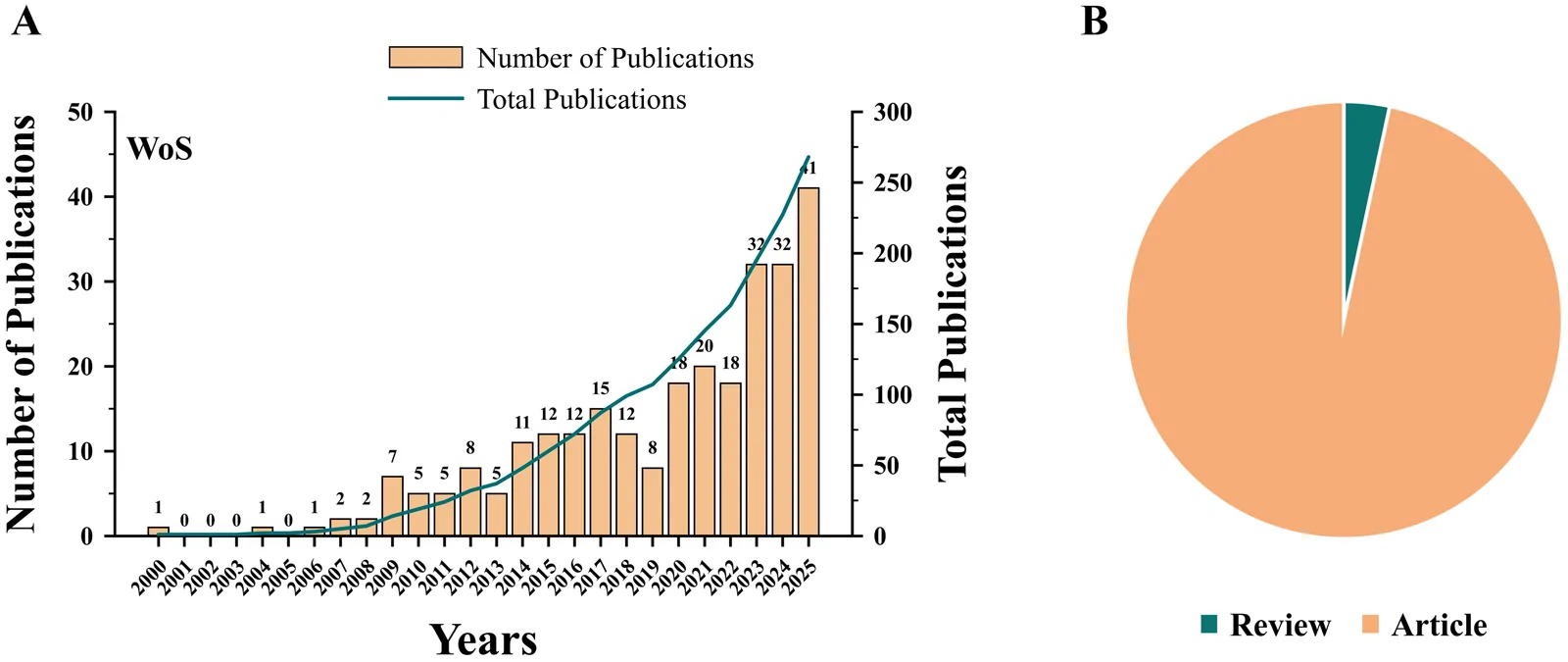
Publication output and document-type distribution of WoSCC records from 2000 to 2025. **(A)** Annual publication output and cumulative number of publications in the WoSCC dataset. Bars represent the number of publications per year, and the line represents total publications. **(B)** Distribution of document types among the included WoSCC records, including articles and reviews.

The initial screening and subsequent verification were concordant for 1,564 of 1,595 records (98.1%), with a descriptive Cohen’s κ of 0.93. Thirty-one binary inclusion/exclusion decisions differed between the two screening rounds and were revised during verification. The final WoSCC dataset comprised 268 publications published between 2000 and 2025 (**Figure 1**). As shown in **Figure 2A**, annual publication output remained very low before 2008 and increased gradually after 2009. A more pronounced increase was observed after 2022, with 32 publications in 2023, 32 in 2024, and 41 in 2025. The cumulative number of publications increased steadily over the study period and reached 268 by the end of 2025. Regarding document type, the dataset included 259 articles and 9 reviews (**Figure 2B**). These results indicate that research on immune-inflammatory biomarkers and biomarker-related immune features in MASLD/NAFLD has expanded substantially in recent years. The top 10 journals ranked by total citations are summarized in **Table 1**. Frontiers in Immunology contributed the largest number of publications (n = 12) and had the highest total citation count (572 citations), followed by Journal of Inflammation-London and Journal of Hepatology. Although several journals contributed only one or a few publications, some showed high average citation counts, including Hepatology, Cell Reports, Nutrition Research, and Journal of Immunology. These results suggest that highly cited studies in this field were distributed across both liver-specialty journals and broader immunology, inflammation, and biomedical journals.

**Table 1:** Top 10 journals by total citations.

Rank	Journal	Documents	IF (2024)	Citations	Average citation
1	Frontiers in Immunology	12	5.9	572	47.67
2	Journal of Inflammation-London	2	4.1	432	216.00
3	Journal of Hepatology	5	33.0	386	77.20
4	Hepatology	1	16.8	374	374.00
5	European Journal of Gastroenterology & Hepatology	4	1.8	340	85.00
6	Cell Reports	1	6.9	284	284.00
7	Nutrition Research	1	3.1	282	282.00
8	Journal of Immunology	1	3.4	270	270.00
9	Gut	3	26.2	252	84.00
10	American Journal of Pathology	1	3.6	244	244.00

### 3.2 Countries, institutions, and authors

**Figure 3:**
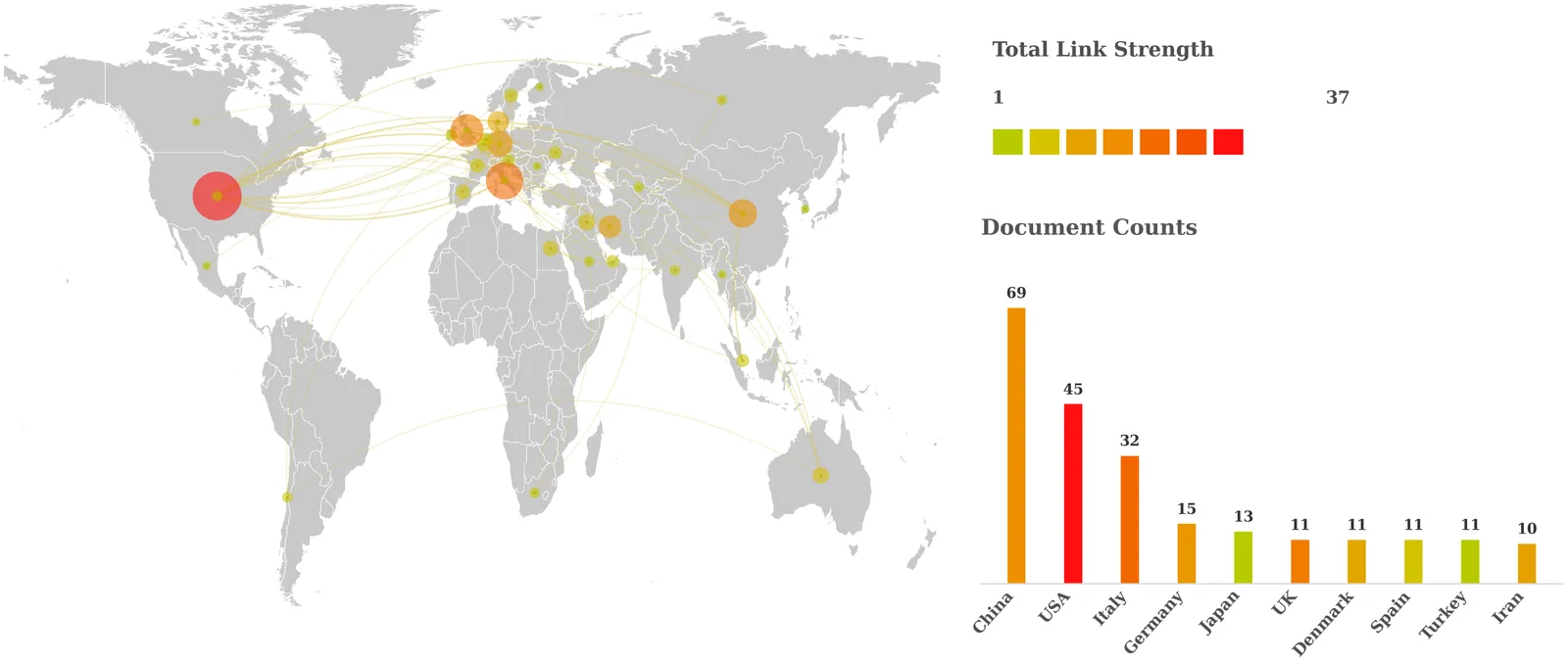
Country-level publication output and international collaboration network. Node size represents publication output, links indicate international collaboration, and color intensity reflects total link strength. The bar chart shows the top 10 contributing countries by document count.

Country-level analysis showed that publications were contributed by multiple countries, with China, the United States, and Italy being the three leading contributors (**Figure 3**). China ranked first with 69 publications, followed by the United States with 45 and Italy with 32. Germany ranked fourth with 15 publications, followed by Japan with 13. The United Kingdom, Denmark, Spain, and Turkey each contributed 11 publications, and Iran contributed 10. The international collaboration map indicated that research collaboration was mainly concentrated among North America, Europe, and East Asia. Although China had the highest publication output, the United States showed the strongest total link strength, suggesting that its international collaborative connections were more extensive relative to its publication count.

**Figure 4:**
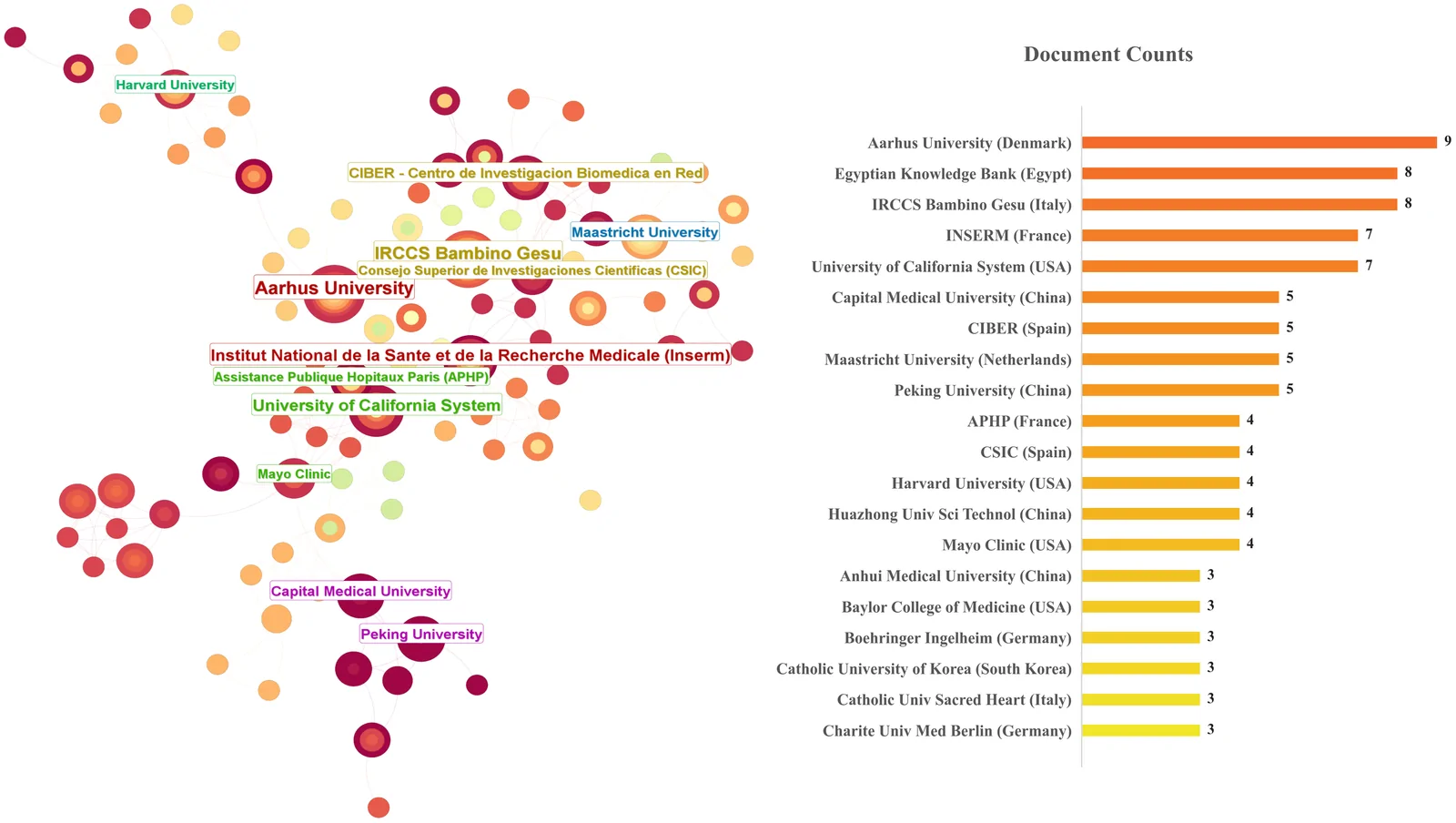
Institutional collaboration network and publication output. The left panel shows the collaboration network among institutions contributing to research on immune-inflammatory biomarkers and immune features in MASLD/NAFLD. The right panel shows the top 20 institutions ranked by document count.

Institutional analysis further showed that contributions were distributed across several European, American, and Asian institutions (**Figure 4**). Aarhus University ranked first with 9 publications, followed by the Egyptian Knowledge Bank and IRCCS Bambino Gesù with 8 publications each. INSERM and the University of California System each contributed 7 publications, while Capital Medical University, CIBER, Maastricht University, and Peking University each contributed 5 publications. The institutional collaboration network showed several visible collaborative groups, including European institutions such as Aarhus University, INSERM, IRCCS Bambino Gesù, Maastricht University, CIBER, and CSIC, as well as Chinese institutions such as Capital Medical University and Peking University.

The top 10 authors by publication output are listed in **Table 2**. Henning Grønbæk and Konstantin Kazankov ranked first, each with 6 publications, both from Aarhus University Hospital in Denmark. Valerio Nobili from Bambino Gesù Pediatric Hospital ranked third with 5 publications, followed by Holger Jon Møller and Anna Alisi. In terms of citation impact, Christopher P. Day had the highest total citations and average citations per document among the top 10 authors, despite contributing 3 publications. Ann Driessen and Sander S. Rensen also showed high average citation counts. These findings suggest that author-level influence in this field was not determined solely by publication volume, as some authors with fewer publications had higher citation impact.

**Table 2:** Top 10 authors by publication output.

No.	Author	Affiliation	Country	Documents		Citations	Average citations	Total link strength
1	Henning Grønbæk	Aarhus University Hospital	Denmark		6	196	32.67	14
2	Konstantin Kazankov	Aarhus University Hospital	Denmark		6	196	32.67	14
3	Valerio Nobili	Bambino Gesù Pediatric Hospital	Italy		5	215	43.00	17
4	Holger Jon Møller	Aarhus University Hospital	Denmark		5	187	37.40	10
5	Anna Alisi	Bambino Gesù Pediatric Hospital	Italy		5	179	35.80	18
6	Emanuele Albano	University of Eastern Piedmont	Italy		4	197	49.25	10
7	Salvatore Sutti	University of Turin	Italy		4	197	49.25	10
8	Christopher P. Day	Newcastle University	United Kingdom		3	598	199.33	0
9	Ann Driessen	Maastricht University Medical Center	Netherlands		3	413	137.67	8
10	Sander S. Rensen	Maastricht University Medical Center	Netherlands		3	396	132.00	6

### 3.3 Keyword analysis

**Figure 5:**
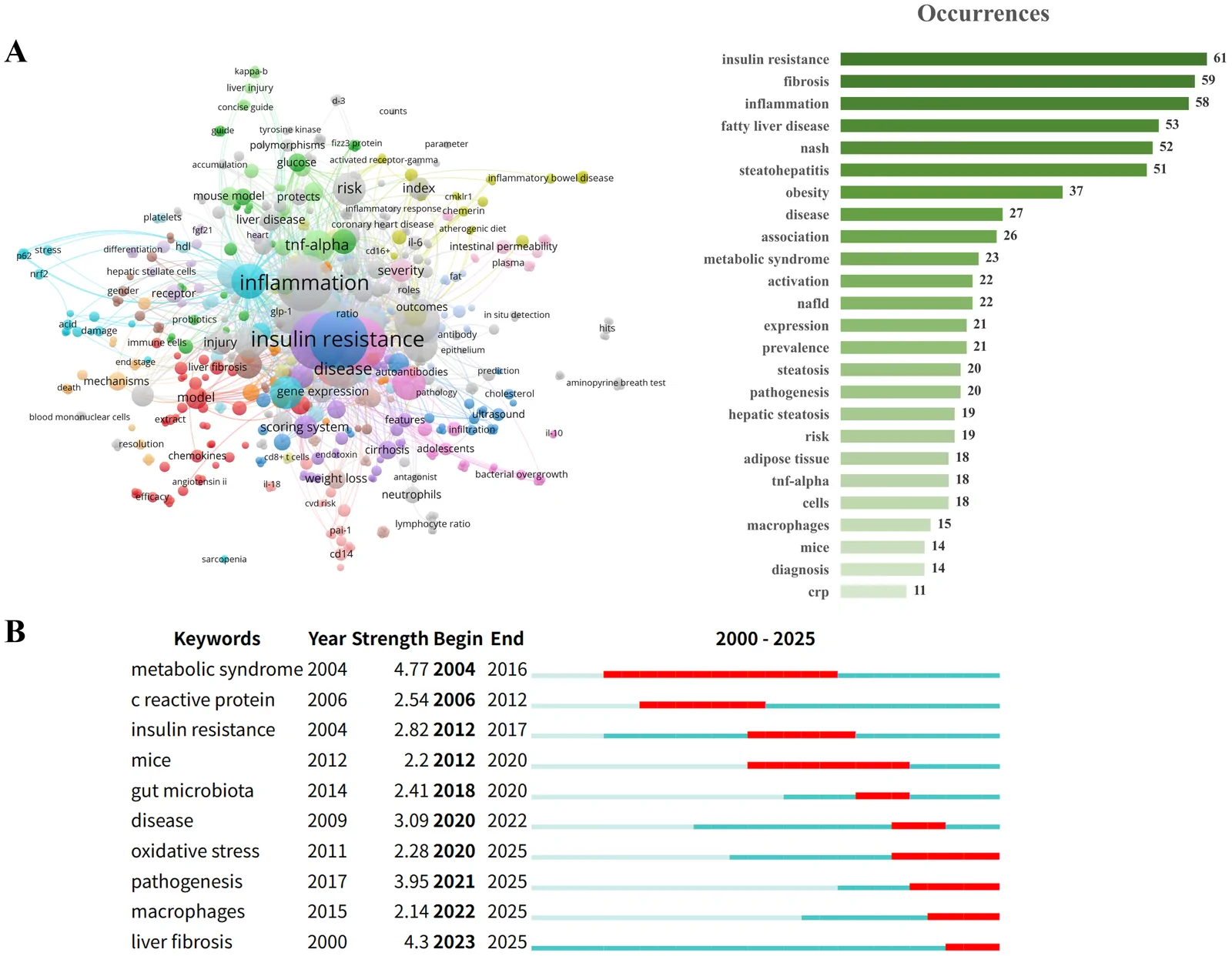
Keyword co-occurrence network and keyword burst analysis in the WoSCC dataset. **(A)** Keyword co-occurrence network and the top keywords by occurrence. The bar chart shows the most frequently occurring keywords. **(B)** Top burst keywords from 2000 to 2025. Red bars indicate the time periods during which each keyword showed a strong keyword burst.

Keyword analysis was performed to identify major research hotspots and emerging trends in this field. In **Figure 5A**, each keyword is represented by a node, and the links between nodes indicate co-occurrence relationships. Keywords that are closer to each other and more densely connected reflect stronger thematic associations. The most frequently occurring keywords were “insulin resistance” (61 occurrences), “fibrosis” (59), “inflammation” (58), “fatty liver disease” (53), “NASH” (52), and “steatohepatitis” (51), followed by “obesity” (37), “disease” (27), “association” (26), and “metabolic syndrome” (23). Other commonly occurring terms included “activation”, “NAFLD”, “expression”, “prevalence”, “steatosis”, “pathogenesis”, “hepatic steatosis”, “risk”, “adipose tissue”, “TNF-alpha”, “cells”, “macrophages”, “mice”, “diagnosis”, and “CRP”. Overall, these keywords indicate that the knowledge base of this field has been centered on metabolic dysfunction, inflammatory activity, fibrosis, and disease progression.

Keyword burst analysis further revealed changes in research attention over time (**Figure 5B**). The strongest and earliest burst keyword was “metabolic syndrome” (strength = 4.77, 2004–2016), followed by “c reactive protein” (strength = 2.54, 2006–2012) and “insulin resistance” (strength = 2.82, 2012–2017). In more recent years, burst keywords shifted toward “oxidative stress” (2020–2025), “pathogenesis” (2021–2025), “macrophages” (2022–2025), and “liver fibrosis” (2023–2025). Among these, “liver fibrosis” showed a particularly strong recent burst (strength = 4.30), suggesting that fibrosis-related assessment has become a major current focus. Together, the co-occurrence and burst results suggest that research in this field has evolved from early emphasis on metabolic syndrome and systemic inflammatory markers toward growing attention to mechanistic processes, macrophage-related immune features, oxidative stress, and fibrosis.

### 3.4 Co-cited references analysis

**Figure 6:**
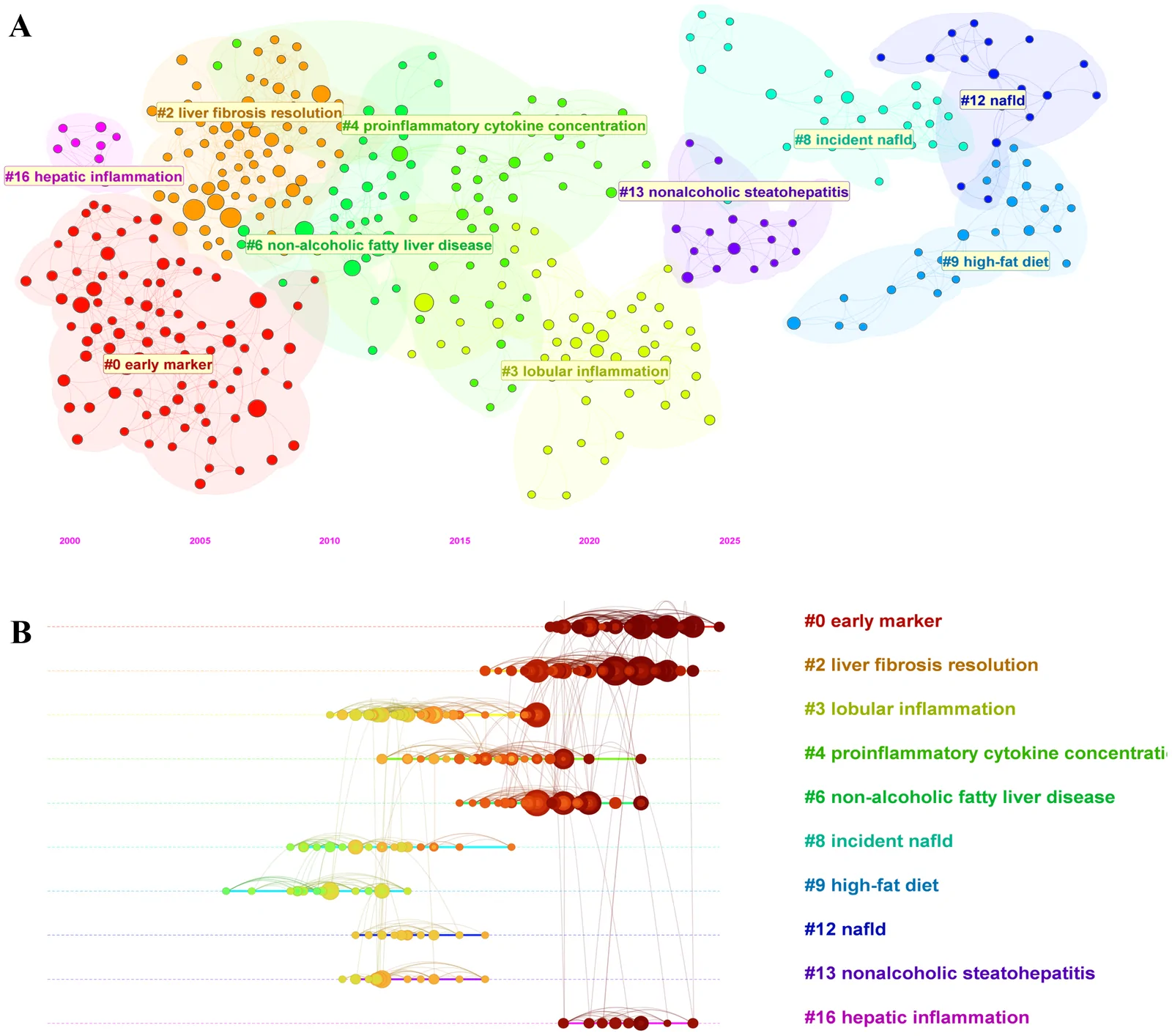
Co-cited reference clustering and timeline visualization in the WoSCC dataset. **(A)** Co-cited reference cluster network labeled using the LSI method. Each node represents a co-cited reference, and clusters indicate groups of references with closely related citation patterns. **(B)** Timeline view of the co-cited reference clusters, showing the temporal distribution of co-cited references across major clusters from 2000 to 2025.

Co-cited reference analysis was performed to characterize the intellectual structure of this field. The modularity Q value was 0.8814, indicating a clearly structured clustering pattern (**Figure 6A**). A total of 10 major clusters were identified. Using the LSI labeling method, these clusters were labeled as #0 “early marker”, #2 “liver fibrosis resolution”, #3 “lobular inflammation”, #4 “proinflammatory cytokine concentration”, #6 “non-alcoholic fatty liver disease”, #8 “incident NAFLD”, #9 “high-fat diet”, #12 “NAFLD”, #13 “nonalcoholic steatohepatitis”, #16 “hepatic inflammation”. The non-consecutive cluster numbering reflected the retained major clusters in the co-citation network. The timeline view showed that co-cited references were distributed across both earlier disease-context and experimental-model clusters, including “NAFLD” and “high-fat diet”, and more recent clusters related to “early marker”, “liver fibrosis resolution”, “proinflammatory cytokine concentration”, and “hepatic inflammation” (**Figure 6B**). The co-citation structure suggested that the knowledge base of this field involved clinically accessible inflammatory markers, hepatic inflammatory activity, fibrosis-related assessment.

### 3.5 Cross-database validation with Scopus

**Figure 7:**
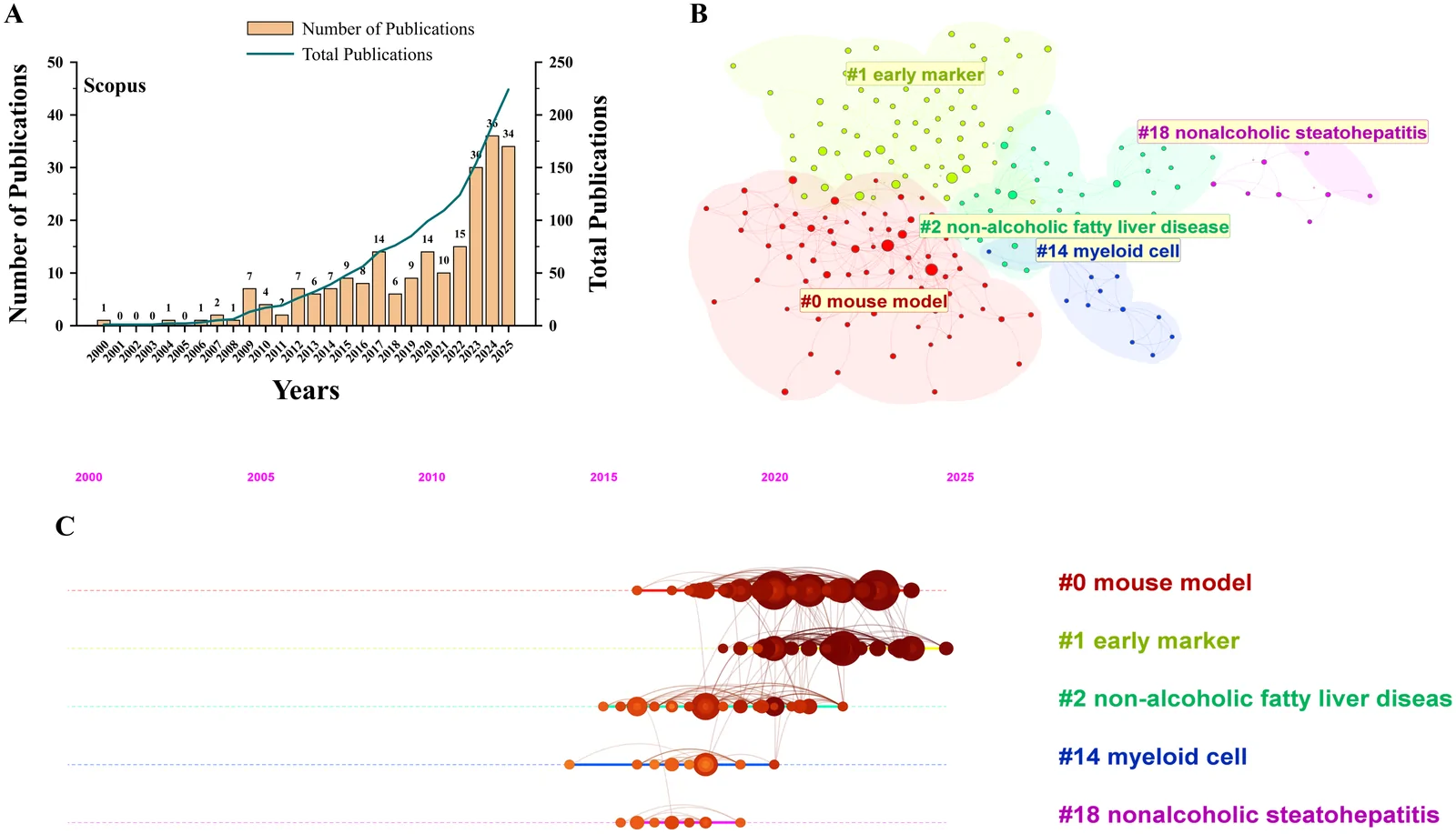
Cross-database validation using the Scopus dataset. **(A)** Annual publication output and cumulative publication trend in the Scopus validation dataset. Bars represent the number of publications per year, and the line represents total publications. **(B)** Co-cited reference cluster network in the Scopus validation dataset. **(C)** Timeline view of Scopus co-cited reference clusters, showing the temporal distribution of co-cited references across the main validation clusters.

To evaluate whether the main findings were dependent on a single bibliographic database, an additional validation analysis was performed using Scopus. The annual publication pattern in the Scopus validation dataset was broadly consistent with that observed in the WoSCC primary dataset, although the absolute number of publications differed between databases. In Scopus, annual output remained low before 2008, increased gradually after 2009, and showed a clear acceleration after 2022, with 30 publications in 2023, 36 in 2024, and 34 in 2025 (**Figure 7A**). This pattern supports the interpretation that the recent expansion of immune-inflammatory biomarker and immune-feature research in MASLD/NAFLD was not an artifact of WoSCC coverage alone.

The Scopus co-cited reference network’s modularity Q value was 0.9094. Compared with the WoSCC co-citation network, the Scopus network produced fewer major clusters, which likely reflects differences in database coverage, citation indexing, and the smaller validation corpus. The main Scopus clusters were labeled as #0 “mouse model”, #1 “early marker”, #2 “non-alcoholic fatty liver disease”, #14 “myeloid cell”, and #18 “nonalcoholic steatohepatitis” (**Figure 7B**). Although the cluster structure was more condensed than that of WoSCC, these themes were consistent with the major knowledge domains identified in the primary analysis, including experimental MASLD/NAFLD models, early biomarker studies, disease-framework research, and immune-cell or myeloid-cell mechanisms.

The Scopus timeline view further showed that co-cited references were concentrated mainly after 2015, with recent activity distributed across clusters related to mouse models, early markers, NAFLD, myeloid cells, and NASH (**Figure 7C**). Taken together, the Scopus validation analysis supported the robustness of the main temporal and thematic conclusions from the WoSCC dataset. However, because the Scopus network yielded fewer clusters, the cross-database comparison should be interpreted at the level of broad research domains rather than exact one-to-one correspondence between individual clusters.

## 4 Discussion

### 4.1 Main bibliometric findings

Research on immune-inflammatory biomarkers and immune features in MASLD/NAFLD has grown quickly, with the sharpest rise after 2022. The Scopus validation dataset showed a similar pattern. China produced the largest number of publications, whereas the United States had the strongest collaborative connectivity. By document count, the leading institutions included Aarhus University, the Egyptian Knowledge Bank, IRCCS Bambino Gesù, INSERM, and the University of California System. At the author level, work by Henning Grønbæk, Konstantin Kazankov, and Holger Jon Møller, was closely linked to macrophage activation biomarkers, especially soluble CD163, and to fibrosis-related assessment (21, 22). Frequent keywords included insulin resistance, fibrosis, inflammation, fatty liver disease, NASH, and steatohepatitis. More recent burst keywords, including oxidative stress, pathogenesis, macrophages, and liver fibrosis, point to growing interest in mechanisms and fibrosis-related biology. The co-citation clusters also showed that the field spans systemic inflammatory indices, hepatic inflammatory activity, macrophage- and immune-cell-related features, fibrosis assessment, treatment-response readouts, and prognostic stratification.

### 4.2 Principal findings and knowledge structure

This study mapped the intellectual structure of research on immune-inflammatory biomarkers and biomarker-related immune features in MASLD/NAFLD. In the Results, the co-citation clusters were reported using their LSI labels, including #0 “early marker”, #2 “liver fibrosis resolution”, #3 “lobular inflammation”, #4 “proinflammatory cytokine concentration”, #6 “non-alcoholic fatty liver disease”, #8 “incident NAFLD”, #9 “high-fat diet”, #12 “NAFLD”, #13 “nonalcoholic steatohepatitis”, #16 “hepatic inflammation”. The cluster structure indicates that the field is described as four overlapping domains: clinically accessible systemic inflammatory biomarkers, hepatic inflammation and histologic injury, immune-cell and macrophage-related features, and fibrosis-, treatment-response-, and prognosis-related readouts.

### 4.3 Accessible systemic inflammatory biomarkers and clinical stratification

Cluster #0 “early marker” and Cluster #12 “NAFLD” mainly represented the clinically accessible biomarker domain. In an NHANES analysis, SII, NLR, and LMR were positively associated with NAFLD, whereas PLR showed an inverted U-shaped relationship (10). A broader analysis found positive associations of SII, SIRI, NLR, and NPAR with MASLD (11). PIV, but not SII, was associated with both NAFLD and fibrosis in another study (23). NHR was associated with NAFLD/MASLD and fibrosis in one analysis (24), but a separate analysis using the same NHANES cycle found an association with MASLD but not fibrosis (25). These differences show that related indices are not interchangeable and that conclusions may depend on outcome definitions, model specification, and subgroup composition.

Meta-analytic evidence was also mixed. NPAR and NAR were higher in patients with NAFLD, but their pooled sensitivity and specificity were moderate (26). Another meta-analysis found higher NLR and lower PLR in NAFLD, with no clear difference in LMR (27). Most individual studies were cross-sectional, whereas a prospective Chinese cohort showed that baseline white blood cell count was associated with incident NAFLD (28). In a smaller high-cardiovascular-risk cohort, insulin sensitivity, postprandial GLP-1, HDL cholesterol, and IFN-γ jointly predicted liver fat (3).

Clinical relevance varied by intended use. IL-6, TNF-α, and hs-CRP were more closely related to metabolic variables than to noninvasive fibrosis severity in an early-stage MASLD cohort (29). hs-CRP showed phenotype-dependent associations with liver fat or stiffness but weak discrimination for MASH or fibrosis (30). For prognosis, NPR was associated with all-cause mortality (31), while NLR and NPAR—but not SII—showed mortality associations in another analysis (32). CLR was associated with NAFLD prevalence but not with all-cause or cardiovascular mortality (33).

The main limitation of this domain are specificity and independence of evidence. Several studies reused overlapping NHANES cycles and recombined the same blood-cell, albumin, CRP, and lipid measurements. These indices may support initial risk enrichment, but they should not be treated as direct measures of hepatic immune activity or as substitutes for established fibrosis assessment without independent prospective validation and evidence of incremental clinical value.

### 4.4 Hepatic inflammation, cytokine activity, and histologic injury

Clusters #3 “lobular inflammation”, #4 “proinflammatory cytokine concentration”, #8 “incident NAFLD”, and #9 “high-fat diet” collectively linked immune-inflammatory markers with liver injury and histologic activity. Hepatic transcriptional profiling identified a NASH-associated immune signature involving antigen presentation, dendritic cells, and cytotoxic cells; intrahepatic CD8 T-cell accumulation was associated with lobular inflammation and ballooning (13). In biopsy-proven NAFLD, IL-6 and IL-10 were related to chronic inflammation and insulin resistance, whereas IL-12 and TNF were more closely associated with liver-damage and fibrosis measures (34). In children, PAI-1, IL-8, soluble IL-2 receptor α, and IL-7 showed different associations with ballooning, lobular inflammation, portal inflammation, and fibrosis (35). Peripheral- and hepatic-vein cytokine ratios also differed in their relationships with metabolic, histologic, and hemodynamic features (36).

The anatomical compartment of inflammation was equally important. Portal infiltrates were dominated by CD68-positive macrophages and CD8-positive lymphocytes and increased with fibrosis and ductular reaction (14). In pediatric NAFLD, activated leukocyte and macrophage markers correlated with histologic severity (37). Obstructive sleep apnea-related hypoxemia was associated with NASH, fibrosis, and intrahepatic inflammatory-cell activation independently of obesity and insulin resistance (38). Adults with NASH showed altered circulating memory T-cell distributions and enhanced IFN-γ production, indicating that peripheral immune changes accompany—but do not necessarily reproduce—the hepatic inflammatory state (39). Serum IgA was associated with advanced fibrosis in a biopsy-based cohort, but it represented a broad immune response rather than a defined hepatic cellular pathway (40).

Several studies explored noninvasive translation. Dynamic FDG-PET parameters were associated with biopsy-defined inflammation, but the pilot cohort included only 22 patients (41). Serum lipocalin-2 correlated with histologic activity and showed high diagnostic estimates for NASH in the evaluated cohort (42). These findings are suggestive, but selected cohorts and limited external validation preclude conclusions about general diagnostic performance. Paired blood–liver measurements and prespecified validation across age, metabolic phenotype, and fibrosis stage are required to determine whether these markers reflect hepatic inflammation rather than systemic metabolic stress.

### 4.5 Immune-cell and macrophage-centered features

Clusters #3 “lobular inflammation” and #13 “nonalcoholic steatohepatitis”, together with immune-cell evidence from #2 “liver fibrosis resolution” and #6 “non-alcoholic fatty liver disease”, represented the immune-cell and macrophage-centered domain. In adults, circulating sCD163 increased with steatohepatitis and advanced fibrosis (43) and was independently associated with morphological disease stage in two cohorts (22). It also decreased after bariatric surgery, particularly among patients with higher baseline disease activity (21). Paired measurements showed that plasma sCD163 increased while hepatic CD163 expression decreased with disease severity, indicating that circulating and tissue measurements are not equivalent (44).

The signal was not consistent across settings. In children with biopsy-proven NAFLD, sCD163 and sMR were poorly associated with histology and did not decline after successful intervention (45). In adults, sCD163 and sMR were associated with fibrosis, but neither improved prediction beyond transient elastography (46). Thus, reproducible biological association does not necessarily translate into incremental clinical value, and adult findings cannot be transferred directly to pediatric disease.

Experimental studies further challenged a binary M1/M2 interpretation. Hepatic p62 accumulation was associated with CD11c-positive macrophages and histologic severity (47). CD11c-positive resident macrophages formed hepatic crown-like structures and promoted fibrosis after hepatocyte death (48). By contrast, fat-laden macrophages expressed inflammatory monocyte markers while also producing IL-10, annexin A1, and other anti-inflammatory mediators (49). Preventing the emergence of a recruited Cx3cr1/Ccr2-expressing macrophage subset reduced crown-like structures but increased fibrosis (9). Macrophage phenotype therefore depends on cellular origin, disease stage, tissue location, and function rather than on a single surface marker.

Single-cell and functional profiling refined this heterogeneity. Liver and bone-marrow myeloid cells acquired a distinct inflammatory phenotype during steatohepatitis (50), while Kupffer cells and monocyte-derived macrophages showed different expression programs across NAFLD stages (51). Peripheral single-cell studies identified changes in immature B cells and myeloid suppressor cells (52), and mass cytometry showed stage-related changes across T-, B-, NK-, and myeloid-cell populations (7). Circulating monocytes from patients with MASH also displayed increased glycolytic and mitochondrial activity (53).

Immune-cell effects remained model-dependent. Depletion of NKT or CD8 T cells reduced disease activity and fibrosis in one dietary model (54), whereas NKT cells showed sex-dependent protective effects in another strain (55). ICOS/ICOSL signaling linked CD8 T cells with TREM2-positive monocyte-derived macrophages (56), while myeloid mineralocorticoid-receptor deficiency reduced steatosis partly by impairing CD8 T-cell activation (57). These discrepancies limit direct extrapolation from a single animal model and support cross-model confirmation followed by validation in human tissue or longitudinal samples.

### 4.6 Fibrosis, treatment response, and prognostic stratification

Clusters #2 “liver fibrosis resolution”, #6 “non-alcoholic fatty liver disease”, and #16 “hepatic inflammation” connected immune-inflammatory features with fibrosis assessment, treatment-response evaluation, and outcome stratification. After bariatric surgery, decreased monocyte PLIN2 was associated with MASH improvement or resolution, whereas increased RAB14 was associated with fibrosis improvement; both outperformed CK18 in the studied cohort (58). In the FALCON 1 analysis, pegbelfermin improved several blood- and imaging-based biomarkers, but agreement with histologic response differed across steatosis, tissue injury, inflammation, and fibrosis and was clearest for steatosis-related measures (59). A Mediterranean-diet intervention improved inflammatory and oxidative-stress markers alongside intrahepatic fat (60). In adolescents undergoing sleeve gastrectomy, liver and visceral adipose tissue showed histologic and macrophage-profile changes, but the study was small and did not establish a general response marker (61).

Experimental studies provided mechanistic context. Combined CCR2/CCR5 inhibition and FGF21 agonism reduced inflammatory monocyte recruitment and improved steatohepatitis and fibrosis in mice (62). A long-acting FGF21 analogue attenuated fibrosis through an NR4A1-mediated Ly6C macrophage switch and altered macrophage–stellate-cell crosstalk (63). SGLT2 inhibitors suppressed PFKFB3-dependent macrophage glycolysis and shifted macrophage polarization in experimental models (64). Myeloid FoxO1 deletion reduced hepatic inflammation, steatosis, and fibrosis (65). These studies identify plausible therapeutic pathways, but a mechanistic target, a pharmacodynamic marker, and a clinically useful response biomarker are not equivalent.

Prognostic evidence within these clusters was limited but clinically relevant. In a multicenter biopsy-proven MASLD cohort, grade 2–3 lobular inflammation and stage 3–4 fibrosis were associated with mortality, and the poorest prognostic profile combined advanced inflammation, ballooning, and fibrosis (66). Biomarkers intended for treatment monitoring or prognosis therefore require validation against use-specific endpoints rather than extrapolation from cross-sectional disease associations.

**Figure 8:**
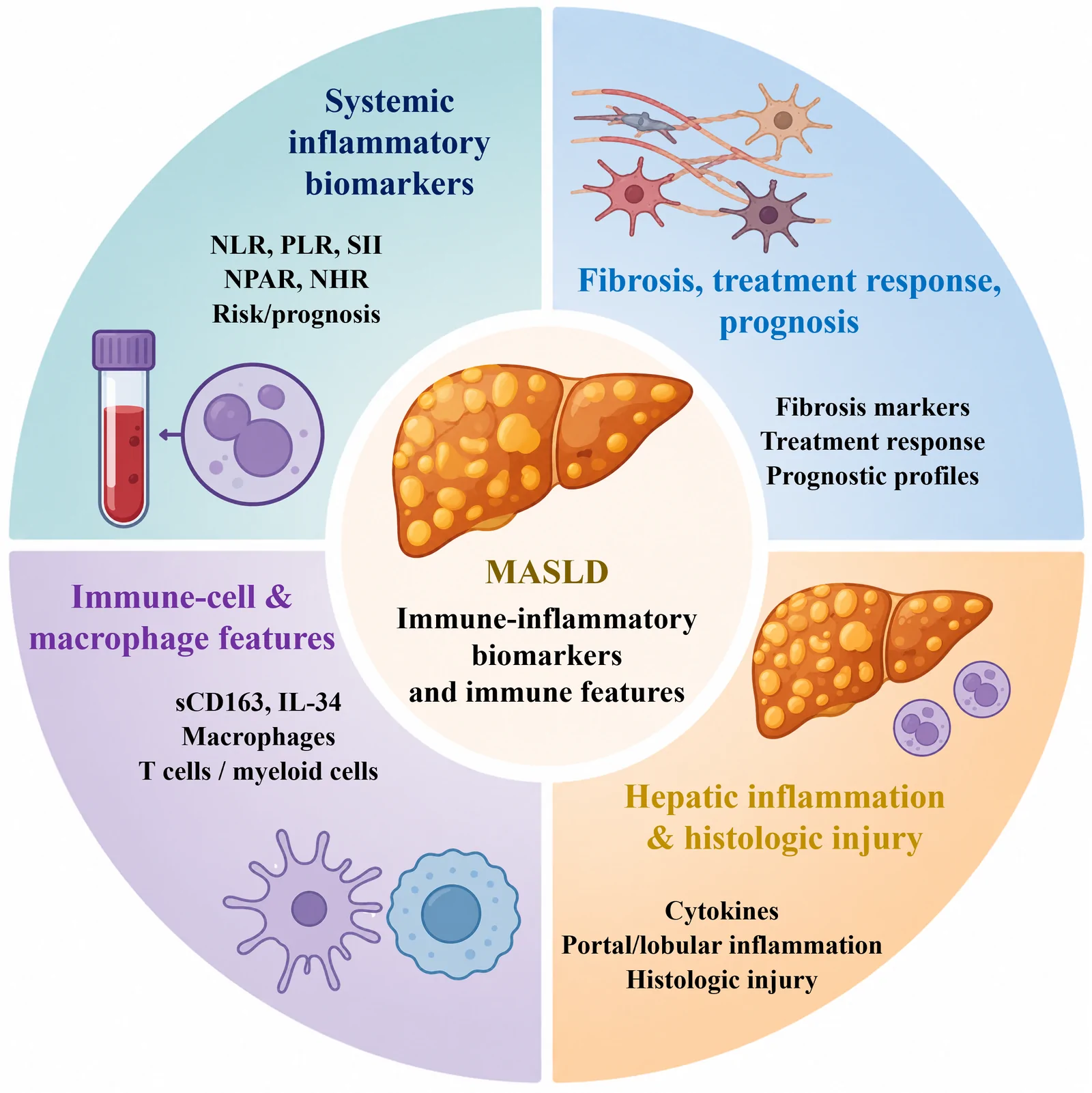
Conceptual framework of major research domains in immune-inflammatory biomarker and immune-feature research in MASLD. Four interconnected research domains synthesized from the bibliometric analysis: systemic inflammatory biomarkers, hepatic inflammation and histologic injury, immune-cell and macrophage features, and fibrosis-, treatment-response-, and prognosis-related readouts. These domains represent overlapping research directions rather than a linear disease pathway or causal mechanism. MASLD, metabolic dysfunction-associated steatotic liver disease; NAFLD, nonalcoholic fatty liver disease; NLR, neutrophil-to-lymphocyte ratio; PLR, platelet-to-lymphocyte ratio; SII, systemic immune-inflammation index; NPAR, neutrophil percentage-to-albumin ratio; NHR, neutrophil-to-high-density lipoprotein cholesterol ratio; sCD163, soluble cluster of differentiation 163; IL-34, interleukin-34.

Taken together, the field has progressed from identifying broad inflammatory associations toward resolving disease-relevant immune states, but this mechanistic refinement has not yet been matched by clinical validation. The central challenge is therefore not the discovery of additional candidate markers, but the establishment of a translational pathway that links a defined hepatic immune process to a scalable and analytically reliable assay, demonstrates incremental value beyond established assessments, and is validated against endpoints appropriate to its intended clinical use. To synthesize these findings, we constructed a conceptual framework summarizing the four major research domains identified in this bibliometric analysis (**Figure 8**).

## 5 Conclusion

This bibliometric study shows that research on immune-inflammatory biomarkers and immune features in MASLD/NAFLD has expanded substantially in recent years. The field is best characterized by several overlapping research domains, including accessible systemic inflammatory indices, hepatic inflammation and histologic injury, immune-cell and macrophage-centered features, fibrosis assessment, treatment-response readouts, and prognostic stratification. Across these domains, a consistent translational gap remains: accessible systemic indices are scalable but often lack hepatic immune specificity, whereas tissue and single-cell features offer greater mechanistic resolution but remain difficult to deploy and insufficiently validated. Current immune-inflammatory biomarkers should therefore be regarded as complementary tools for risk stratification, phenotype refinement, and mechanistic interpretation rather than substitutes for established histologic, elastographic, or imaging-based assessment. Future progress will require moving beyond candidate discovery toward use-specific validation pathways that link defined immune processes to analytically reliable assays, demonstrate incremental value beyond established assessments, and validate performance against clinically relevant endpoints in prospective cohorts, treatment-response settings, and relevant patient subgroups.

## 6 Research limitations

Several limitations should be considered. First, WoSCC was used as the primary bibliometric dataset, although Scopus was included for cross-database validation. Because the analysis was restricted to English-language articles and reviews, relevant studies indexed in other databases, published in other languages, or classified as other document types may have been missed. Second, citation-based analyses are affected by citation lag, which may underestimate the influence of recently published studies. Third, author and institutional name disambiguation remains imperfect in bibliometric analyses and may affect collaboration and productivity estimates. Fourth, cluster labels are partly algorithm-dependent, and the transition from NAFLD/NASH to MASLD/MASH terminology may have dispersed semantically related citation streams. Finally, the biomarker literature itself remains heterogeneous: markers are not fully standardized, studies use different endpoints such as disease presence, inflammatory activity, fibrosis stage, treatment response, or prognosis, and mechanistic findings are not yet consistently linked to clinically deployable assays. In addition, the observed research landscape is shaped not only by scientific interest but also by data and technical accessibility: routine systemic inflammatory indices can be readily examined in public datasets, whereas tissue-based and single-cell studies face greater sampling and analytical barriers. Consequently, accessible topics may be overrepresented and technically demanding topics underrepresented, creating a potential mismatch between publication volume and the underlying biological or clinical importance of different research domains.

## Data Availability

Publicly available datasets were analyzed in this study. This data can be found here: https://www.webofscience.com/ & https://www.scopus.com/.

## References

[1] Rinella ME Lazarus JV Ratziu V Francque SM Sanyal AJ Kanwal F . A multisociety delphi consensus statement on new fatty liver disease nomenclature. J Hepatol. (2023) 79:1542–56. doi: 10.1016/j.jhep.2023.06.003 37364790

[2] Younossi ZM Golabi P Paik JM Henry A Van Dongen C Henry L . The global epidemiology of nonalcoholic fatty liver disease (NAFLD) and nonalcoholic steatohepatitis (NASH): a systematic review. Hepatology. (2023) 77:1335–47. doi: 10.1097/HEP.0000000000000004 36626630 PMC10026948

[3] Bozzetto L Annuzzi G Ragucci M Di Donato O Della Pepa G Della Corte G . Insulin resistance, postprandial GLP-1 and adaptive immunity are the main predictors of NAFLD in a homogeneous population at high cardiovascular risk. Nutr Metab Cardiovasc Dis NMCD. (2016) 26:623–9. doi: 10.1016/j.numecd.2016.01.011 27134062

[4] Lambrecht J Tacke F . Controversies and opportunities in the use of inflammatory markers for diagnosis or risk prediction in fatty liver disease. Front Immunol. (2020) 11:634409. doi: 10.3389/fimmu.2020.634409 33633748 PMC7900147

[5] Loomba R Mohseni R Lucas KJ Gutierrez JA Perry RG Trotter JF . TVB-2640 (FASN inhibitor) for the treatment of nonalcoholic steatohepatitis: FASCINATE-1, a randomized, placebo-controlled phase 2a trial. Gastroenterology. (2021) 161:1475–86. doi: 10.1053/j.gastro.2021.07.025 34310978

[6] Forlano R Martinez-Gili L Takis P Miguens-Blanco J Liu T Triantafyllou E . Disruption of gut barrier integrity and host–microbiome interactions underlie MASLD severity in patients with type-2 diabetes mellitus. Gut Microbes. (2024) 16:2304157. doi: 10.1080/19490976.2024.2304157 38235661 PMC10798360

[7] Waller KJ Saihi H Li W Brindley JH De Jong A Syn W-K . Single-cell phenotypes of peripheral blood immune cells in early and late stages of non-alcoholic fatty liver disease. Clin Mol Hepatol. (2023) 29:417–32. doi: 10.3350/cmh.2022.0205 36727210 PMC10121278

[8] Laursen TL Mellemkjær A Møller HJ Grønbæk H Kazankov K . Spotlight on liver macrophages for halting injury and progression in nonalcoholic fatty liver disease. Expert Opin Ther Targets. (2022) 26:697–705. doi: 10.1080/14728222.2022.2132145 36205054

[9] Daemen S Gainullina A Kalugotla G He L Chan MM Beals JW . Dynamic shifts in the composition of resident and recruited macrophages influence tissue remodeling in NASH. Cell Rep. (2021) 34:108626. doi: 10.1016/j.celrep.2020.108626 33440159 PMC7877246

[10] Liu K Tang S Liu C Ma J Cao X Yang X . Systemic immune-inflammatory biomarkers (SII, NLR, PLR and LMR) linked to non-alcoholic fatty liver disease risk. Front Immunol. (2024) 15:1337241. doi: 10.3389/fimmu.2024.1337241 38481995 PMC10933001

[11] Wang Y Chen S Tian C Wang Q Yang Z Che W . Association of systemic immune biomarkers with metabolic dysfunction-associated steatotic liver disease: a cross-sectional study of NHANES 2007-2018. Front Nutr. (2024) 11:1415484. doi: 10.3389/fnut.2024.1415484 39296508 PMC11408230

[12] Wang Y Huang DQ Zhang P Wang M Wu Y Nur E . Plasma inflammatory proteome profiles identify MASLD among children with overweight or obesity. Cardiovasc Diabetol. (2025) 24:450. doi: 10.1186/s12933-025-03011-0 41310718 PMC12659286

[13] Haas JT Vonghia L Mogilenko DA Verrijken A Molendi-Coste O Fleury S . Transcriptional network analysis implicates altered hepatic immune function in NASH development and resolution. Nat Metab. (2019) 1:604–14. doi: 10.1038/s42255-019-0076-1 31701087 PMC6837876

[14] Gadd VL Skoien R Powell EE Fagan KJ Winterford C Horsfall L . The portal inflammatory infiltrate and ductular reaction in human nonalcoholic fatty liver disease. Hepatology. (2014) 59:1393–405. doi: 10.1002/hep.26937 24254368

[15] Zhang T-S Qin H-L Wang T Li H-T Li H Xia S-H . Global publication trends and research hotspots of nonalcoholic fatty liver disease: a bibliometric analysis and systematic review. SpringerPlus. (2015) 4:776. doi: 10.1186/s40064-015-1542-1 26697286 PMC4678134

[16] Li Z Cao S Zhao S Kang N . A bibliometric analysis and visualization of nonalcoholic fatty liver disease from 2012 to 2021. Clin Exp Med. (2023) 23:1961–71. doi: 10.1007/s10238-023-01023-2 36795238

[17] Yang S Yu D Liu J Qiao Y Gu S Yang R . Global publication trends and research hotspots of the gut-liver axis in NAFLD: a bibliometric analysis. Front Endocrinol. (2023) 14:1121540. doi: 10.3389/fendo.2023.1121540 36967792 PMC10034112

[18] Yang Z Xiong Z Wang Q Zhou N . A bibliometric analysis of macrophages associated with non-alcoholic fatty liver disease research from 2005 to 2023. Heliyon. (2024) 10:e24187. doi: 10.1016/j.heliyon.2024.e24187 38293366 PMC10827458

[19] van Eck NJ Waltman L . Software survey: VOSviewer, a computer program for bibliometric mapping. Scientometrics. (2010) 84:523–38. doi: 10.1007/s11192-009-0146-3 20585380 PMC2883932

[20] Chen C . Searching for intellectual turning points: progressive knowledge domain visualization. Proc Natl Acad Sci USA. (2004) 101 Suppl 1:5303–10. doi: 10.1073/pnas.0307513100 14724295 PMC387312

[21] Kazankov K Tordjman J Møller HJ Vilstrup H Poitou C Bedossa P . Macrophage activation marker soluble CD163 and non-alcoholic fatty liver disease in morbidly obese patients undergoing bariatric surgery. J Gastroenterol Hepatol. (2015) 30:1293–300. doi: 10.1111/jgh.12943 25772748

[22] Kazankov K Barrera F Møller HJ Rosso C Bugianesi E David E . The macrophage activation marker sCD 163 is associated with morphological disease stages in patients with non-alcoholic fatty liver disease. Liver Int. (2016) 36:1549–57. doi: 10.1111/liv.13150 27102725

[23] Jiang R Hua Y Hu X Hong Z . The pan immune inflammatory value in relation to non-alcoholic fatty liver disease and hepatic fibrosis. Clin Res Hepatol Gastroenterol. (2024) 48:102393. doi: 10.1016/j.clinre.2024.102393 38866239

[24] Zhu N Li Y Lin Y Cui X Li X . Association between neutrophil-to-high-density lipoprotein cholesterol ratio and non-alcoholic fatty liver disease or metabolic dysfunction-associated steatotic liver disease: evidence from NHANES 2017–2020. Front Med. (2025) 11:1491858. doi: 10.3389/fmed.2024.1491858 39882525 PMC11774988

[25] Lu Y Xu X Wu J Ji L Huang H Chen M . Association between neutrophil-to-high-density lipoprotein cholesterol ratio and metabolic dysfunction-associated steatotic liver disease and liver fibrosis in the US population: a nationally representative cross-sectional study using NHANES data from 2017 to 2020. BMC Gastroenterol. (2024) 24:300. doi: 10.1186/s12876-024-03394-6 39237899 PMC11378436

[26] El-Sehrawy AAMA Jafari M Zwamel AH Rashidian P Ballal S Kalia R . Neutrophil percentage-to-albumin ratio and neutrophil-to-albumin ratio as novel biomarkers for non-alcoholic fatty liver disease: a systematic review and meta-analysis. J Health Popul Nutr. (2025) 44:167. doi: 10.1186/s41043-025-00926-y 40413495 PMC12102820

[27] Yang Y He X Tan S Qu X Huang W Cai J . The association between immunoinflammatory biomarkers NLR, PLR, LMR and nonalcoholic fatty liver disease: a systematic review and meta-analysis. Clin Exp Med. (2025) 25:39. doi: 10.1007/s10238-024-01539-1 39812894 PMC11735594

[28] Wang S Zhang C Zhang G Yuan Z Liu Y Ding L . Association between white blood cell count and non-alcoholic fatty liver disease in urban han chinese: a prospective cohort study. BMJ Open. (2016) 6:e010342. doi: 10.1136/bmjopen-2015-010342 27251683 PMC4893843

[29] Eid RA Hamed AM Elgendy SO Orayj KM Ibrahim ARN Hamied AMA . Associations between systemic inflammatory markers, metabolic dysfunction, and liver fibrosis scores in patients with MASLD. Metabolites. (2025) 16:25. doi: 10.3390/metabo16010025 41590632 PMC12843941

[30] Wang H Ye J Chen Y Sun Y Gong X Deng H . High sensitivity C-reactive protein implicates heterogeneous metabolic phenotypes and severity in metabolic dysfunction associated-steatotic liver disease. BMC Gastroenterol. (2025) 25:231. doi: 10.1186/s12876-025-03778-2 40200156 PMC11980055

[31] Ye D Ji X Ma Y Shi J Wang J Chen J . NPR is an independent risk factor for predicting all-cause mortality in patients with metabolic dysfunction-associated steatotic liver disease: evidence from NHANES 2007–2020. Popul Health Metrics. (2025) 23:15. doi: 10.1186/s12963-025-00378-w 40281626 PMC12032722

[32] Dong K Zheng Y Wang Y Guo Q . Predictive role of neutrophil percentage-to-albumin ratio, neutrophil-to-lymphocyte ratio, and systemic immune-inflammation index for mortality in patients with MASLD. Sci Rep. (2024) 14:30403. doi: 10.1038/s41598-024-80801-8 39638820 PMC11621551

[33] Xi J Wang S Chen J Law JCS Fan Z Lv G . The role of C-reactive protein to lymphocyte ratio in NAFLD and mortality among NAFLD patients. BMC Gastroenterol. (2025) 25:327. doi: 10.1186/s12876-025-03924-w 40312728 PMC12044991

[34] Fontes-Cal TCM Mattos RT Medeiros NI Pinto BF Belchior-Bezerra M Roque-Souza B . Crosstalk between plasma cytokines, inflammation, and liver damage as a new strategy to monitoring NAFLD progression. Front Immunol. (2021) 12:708959. doi: 10.3389/fimmu.2021.708959 34447378 PMC8383065

[35] Perito ER Ajmera V Bass NM Rosenthal P Lavine JE Schwimmer JB . Association between cytokines and liver histology in children with nonalcoholic fatty liver disease. Hepatol Commun. (2017) 1:609–22. doi: 10.1002/hep4.1068 29130075 PMC5679472

[36] Vonghia L Magrone T Verrijken A Michielsen P Van Gaal L Jirillo E . Peripheral and hepatic vein cytokine levels in correlation with non-alcoholic fatty liver disease (NAFLD)-related metabolic, histological, and haemodynamic features. PloS One. (2015) 10:e0143380. doi: 10.1371/journal.pone.0143380 26599575 PMC4658042

[37] De Vito R Alisi A Masotti A Ceccarelli S Panera N Citti A . Markers of activated inflammatory cells correlate with severity of liver damage in children with nonalcoholic fatty liver disease. Int J Mol Med. (2012) 30:49–56. doi: 10.3892/ijmm.2012.965 22505182

[38] Nobili V Cutrera R Liccardo D Pavone M Devito R Giorgio V . Obstructive sleep apnea syndrome affects liver histology and inflammatory cell activation in pediatric nonalcoholic fatty liver disease, regardless of obesity/insulin resistance. Am J Respir Crit Care Med. (2014) 189:66–76. doi: 10.1164/rccm.201307-1339OC 24256086

[39] Inzaugarat ME Ferreyra Solari NE Billordo LA Abecasis R Gadano AC Cherñavsky AC . Altered phenotype and functionality of circulating immune cells characterize adult patients with nonalcoholic steatohepatitis. J ClinImmunol. (2011) 31:1120–30. doi: 10.1007/s10875-011-9571-1 21845516

[40] McPherson S Henderson E Burt AD Day CP Anstee QM . Serum immunoglobulin levels predict fibrosis in patients with non-alcoholic fatty liver disease. J Hepatol. (2014) 60:1055–62. doi: 10.1016/j.jhep.2014.01.010 24445215

[41] Sarkar S Corwin MT Olson KA Stewart SL Liu C-H Badawi RD . Pilot study to diagnose nonalcoholic steatohepatitis with Dynamic^18^ F-FDG PET. Am J Roentgenol. (2019) 212:529–37. doi: 10.2214/AJR.18.20012 30673340

[42] Xu G Wang Y-M Ying M-M Chen S-D Li Z-R Ma H-L . Serum lipocalin-2 is a potential biomarker for the clinical diagnosis of nonalcoholic steatohepatitis. Clin Mol Hepatol. (2021) 27:329–45. doi: 10.3350/cmh.2020.0261 33465844 PMC8046622

[43] Mueller JL Feeney ER Zheng H Misdraji J Kruger AJ Alatrakchi N . Circulating soluble CD163 is associated with steatohepatitis and advanced fibrosis in nonalcoholic fatty liver disease. Clin Transl Gastroenterol. (2015) 6:e114. doi: 10.1038/ctg.2015.36 26448455 PMC4816035

[44] Skytthe MK Pedersen FB Wernberg CW Indira Chandran V Krag A Di Caterino T . Obese patients with nonalcoholic fatty liver disease have an increase in soluble plasma CD163 and a concurrent decrease in hepatic expression of CD163. Gastro Hep Adv. (2023) 2:711–20. doi: 10.1016/j.gastha.2023.03.006 39129874 PMC11307542

[45] Kazankov K Alisi A Møller HJ De Vito R Rittig S Mahler B . Macrophage markers are poorly associated with liver histology in children with nonalcoholic fatty liver disease. J Pediatr Gastroenterol Nutr. (2018) 67:635–42. doi: 10.1097/MPG.0000000000002111 30074574

[46] Kazankov K Rosso C Younes R Armandi A Hagström H Møller HJ . Macrophage markers do not add to the prediction of liver fibrosis by transient elastography in patients with metabolic associated fatty liver disease. Front Med. (2020) 7:616212. doi: 10.3389/fmed.2020.616212 33392234 PMC7775526

[47] Fukushima H Yamashina S Arakawa A Taniguchi G Aoyama T Uchiyama A . Formation of p62-positive inclusion body is associated with macrophage polarization in non-alcoholic fatty liver disease. Hepatol Res. (2018) 48:757–67. doi: 10.1111/hepr.13071 29473277

[48] Itoh M Suganami T Kato H Kanai S Shirakawa I Sakai T . CD11c+ resident macrophages drive hepatocyte death-triggered liver fibrosis in a murine model of nonalcoholic steatohepatitis. JCI Insight. (2017) 2:e92902. doi: 10.1172/jci.insight.92902 29202448 PMC5752377

[49] Jindal A Bruzzì S Sutti S Locatelli I Bozzola C Paternostro C . Fat-laden macrophages modulate lobular inflammation in nonalcoholic steatohepatitis (NASH). Exp Mol Pathol. (2015) 99:155–62. doi: 10.1016/j.yexmp.2015.06.015 26112094

[50] Krenkel O Hundertmark J Abdallah AT Kohlhepp M Puengel T Roth T . Myeloid cells in liver and bone marrow acquire a functionally distinct inflammatory phenotype during obesity-related steatohepatitis. Gut. (2020) 69:551–63. doi: 10.1136/gutjnl-2019-318382 31076404

[51] Su Q Kim SY Adewale F Zhou Y Aldler C Ni M . Single-cell RNA transcriptome landscape of hepatocytes and non-parenchymal cells in healthy and NAFLD mouse liver. iScience. (2021) 24:103233. doi: 10.1016/j.isci.2021.103233 34755088 PMC8560975

[52] Steixner-Kumar AA Santacruz D Geiger T Rust W Böttner D Krenkel O . Single-cell landscape of peripheral immune cells in MASLD/MASH. Hepatol Commun. (2025) 9(5):e0643. doi: 10.1097/HC9.0000000000000643 40257301 PMC12014121

[53] Sangineto M Ciarnelli M Colangelo T Moola A Bukke VN Duda L . Monocyte bioenergetics: an immunometabolic perspective in metabolic dysfunction-associated steatohepatitis. Cell Rep Med. (2024) 5:101564. doi: 10.1016/j.xcrm.2024.101564 38733988 PMC11148801

[54] Bhattacharjee J Kirby M Softic S Miles L Salazar-Gonzalez R-M Shivakumar P . Hepatic natural killer T-cell and CD8+ T-cell signatures in mice with nonalcoholic steatohepatitis. Hepatol Commun. (2017) 1:299–310. doi: 10.1002/hep4.1041 29152605 PMC5687094

[55] Cuño-Gómiz C De Gregorio E Tutusaus A Rider P Andrés-Sánchez N Colell A . Sex-based differences in natural killer T cell-mediated protection against diet-induced steatohepatitis in balb/c mice. Biol Sex Differ. (2023) 14:85. doi: 10.1186/s13293-023-00569-w 37964320 PMC10644614

[56] Provera A Ramavath NN Gadipudi LL Gigliotti CL Boggio E Vecchio C . Role of the co-stimulatory molecule inducible T-cell co-stimulator ligand (ICOSL) in the progression of experimental metabolic dysfunction-associated steatohepatitis. Front Immunol. (2023) 14:1290391. doi: 10.3389/fimmu.2023.1290391 38077334 PMC10702974

[57] Muñoz-Durango N Arrese M Hernández A Jara E Kalergis AM Cabrera D . A mineralocorticoid receptor deficiency in myeloid cells reduces liver steatosis by impairing activation of CD8+ T cells in a nonalcoholic steatohepatitis mouse model. Front Immunol. (2020) 11:563434. doi: 10.3389/fimmu.2020.563434 33391254 PMC7772468

[58] Angelini G Panunzi S Pompili M Riccardi L Garcovich M Verrastro O . Performance of noninvasive tests for metabolic dysfunction-associated steatohepatitis and liver fibrosis resolution after bariatric surgery. Clin Chem. (2025) 71:406–17. doi: 10.1093/clinchem/hvae208 39786442

[59] Brown EA Minnich A Sanyal AJ Loomba R Du S Schwarz J . Effect of pegbelfermin on NASH and fibrosis-related biomarkers and correlation with histological response in the FALCON 1 trial. JHEP Rep. (2023) 5:100661. doi: 10.1016/j.jhepr.2022.100661 36866389 PMC9971179

[60] Quetglas-Llabrés MM Monserrat-Mesquida M Bouzas C Llompart I Mateos D Casares M . Mediterranean diet improves plasma biomarkers related to oxidative stress and inflammatory process in patients with non-alcoholic fatty liver disease. Antioxidants. (2023) 12:833. doi: 10.3390/antiox12040833 37107208 PMC10134978

[61] Franchitto A Carpino G Alisi A De Peppo F Overi D De Stefanis C . The contribution of the adipose tissue-liver axis in pediatric patients with nonalcoholic fatty liver disease after laparoscopic sleeve gastrectomy. J Pediatr. (2020) 216:117–127.e2. doi: 10.1016/j.jpeds.2019.07.037 31526528

[62] Puengel T Lefere S Hundertmark J Kohlhepp M Penners C Van De Velde F . Combined therapy with a CCR2/CCR5 antagonist and FGF21 analogue synergizes in ameliorating steatohepatitis and fibrosis. Int J Mol Sci. (2022) 23:6696. doi: 10.3390/ijms23126696 35743140 PMC9224277

[63] Ji Y Duan Y Li Y Lu Q Liu D Yang Y . A long-acting FGF21 attenuates metabolic dysfunction-associated steatohepatitis-related fibrosis by modulating NR4A1-mediated Ly6C phenotypic switch in macrophages. Br J Pharmacol. (2024) 181:2923–46. doi: 10.1111/bph.16378 38679486

[64] Lin X-F Cui X-N Yang J Jiang Y-F Wei T-J Xia L . SGLT2 inhibitors ameliorate NAFLD in mice via downregulating PFKFB3, suppressing glycolysis and modulating macrophage polarization. Acta Pharmacol Sin. (2024) 45:2579–97. doi: 10.1038/s41401-024-01389-3 39294445 PMC11579449

[65] Lee S Usman TO Yamauchi J Chhetri G Wang X Coudriet GM . Myeloid FoxO1 depletion attenuates hepatic inflammation and prevents nonalcoholic steatohepatitis. J Clin Invest. (2022) 132:e154333. doi: 10.1172/JCI154333 35700043 PMC9282937

[66] Tsutsumi T Kawaguchi T Fujii H Kamada Y Takahashi H Kawanaka M . Hepatic inflammation and fibrosis are profiles related to mid-term mortality in biopsy-proven MASLD: a multicenter study in Japan. Aliment Pharmacol Ther. (2024) 59:1559–70. doi: 10.1111/apt.17995 38651312

